# Bacterial extracellular electron transfer: a powerful route to the green biosynthesis of inorganic nanomaterials for multifunctional applications

**DOI:** 10.1186/s12951-021-00868-7

**Published:** 2021-04-27

**Authors:** Long Zou, Fei Zhu, Zhong-er Long, Yunhong Huang

**Affiliations:** Nanchang Key Laboratory of Microbial Resources Exploitation & Utilization From Poyang Lake Wetland, College of Life Sciences, Jiangxi Normal University, Nanchang, 330022 China

**Keywords:** Extracellular electron transfer, Biosynthesis, Inorganic nanomaterials, Microbial nano-factory, Metal nanoparticles

## Abstract

Synthesis of inorganic nanomaterials such as metal nanoparticles (MNPs) using various biological entities as smart nanofactories has emerged as one of the foremost scientific endeavors in recent years. The biosynthesis process is environmentally friendly, cost-effective and easy to be scaled up, and can also bring neat features to products such as high dispersity and biocompatibility. However, the biomanufacturing of inorganic nanomaterials is still at the trial-and-error stage due to the lack of understanding for underlying mechanism. Dissimilatory metal reduction bacteria, especially *Shewanella* and *Geobacter* species, possess peculiar extracellular electron transfer (EET) features, through which the bacteria can pump electrons out of their cells to drive extracellular reduction reactions, and have thus exhibited distinct advantages in controllable and tailorable fabrication of inorganic nanomaterials including MNPs and graphene. Our aim is to present a critical review of recent state-of-the-art advances in inorganic biosynthesis methodologies based on bacterial EET using *Shewanella* and *Geobacter* species as typical strains. We begin with a brief introduction about bacterial EET mechanism, followed by reviewing key examples from literatures that exemplify the powerful activities of EET-enabled biosynthesis routes towards the production of a series of inorganic nanomaterials and place a special emphasis on rationally tailoring the structures and properties of products through the fine control of EET pathways. The application prospects of biogenic nanomaterials are then highlighted in multiple fields of (bio-) energy conversion, remediation of organic pollutants and toxic metals, and biomedicine. A summary and outlook are given with discussion on challenges of bio-manufacturing with well-defined controllability.

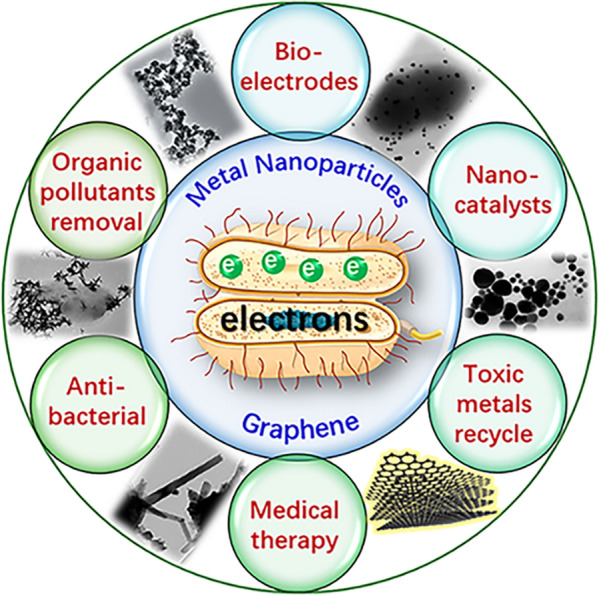

## Introduction

Nanostructured materials having at least one of their dimension sizes smaller than 100 nm have demonstrated wide applicability in producing industrial products and daily necessities. The fabrication and utilization of nanomaterials have thus sparked widespread interest from both academia and industries. One such important class of nanomaterials that have allured global researchers is metal nanoparticles (MNPs), which have become crucial components in multiple cutting-edge areas including catalysis, sensors, clinical diagnosis, nanomedicine, antimicrobial agents, environmental remediation and agriculture [1–4].

Two categories of nanofabrication technologies are known as top-down and bottom-up approaches [5]. For the former, nanosized materials are prepared through the rupture of bulk materials to fine particles, and such a process is usually conducted by diverse physical and mechanical techniques like lithography, laser ablation, sputtering, ball milling and arc-discharging [6, 7]. These techniques themselves are simple, and nanosized materials can be produced quickly after relatively short technological process, but expensive specialized equipment and high energy consumption are usually inevitable. Meanwhile, a variety of efficient chemical bottom-up methods, where atoms assemble into nuclei and then form nanoparticles, have been intensively studied to synthesize and modulate nanomaterials with specific shape and size [8].

Indeed, chemical methodologies, including but not limited to, aqueous reaction using chemical reducing agents (e.g. hydrazine hydrate and sodium borohydride), electrochemical deposition, hydrothermal/solvothermal synthesis, sol–gel processing, chemical liquid/vapor deposition, have been developed up to now [5, 6]. These approaches can not only produce diverse nanomaterials with fairly high yields, but also endow fine controllability in tailoring nanostructures and properties of the products. Nevertheless, they have been encountering some serious challenges of harsh reaction conditions (e.g. pH and temperature), potential risks in human health and environment, and low cost-effectiveness. Moreover, there are biosafety concerns on products synthesized chemically using hazardous reagents, which restricts their applications in many areas, particularly in medicines and pharmaceuticals [9].

Impressively, biological methodology is becoming a favourite in nanomaterial synthesis nowadays to address challenges in chemical synthesis. Compared to chemical routes, biosynthesis using natural and biological materials as reducing, stabilizing and capping agents are simple, energy- and cost-effective, mild and environment-friendly, which is termed as “Green Chemistry” [2, 6]. More significantly, the biologically synthesized nanomaterials have much better competitiveness in biocompatibility, compared to those chemically derived counterparts. On the one hand, the biogenic nanomaterials are free from toxic contamination of by-products that are usually involved in chemical synthesis process; on the other hand, the biosynthesis do not need additional stabilizing agents because either the used organisms themselves or their constituents can act as capping and stabilizing agents and the attached biological components in turn form biocompatible envelopes on the resultant nanomaterials, leading to actively interact with biological systems [2]. As one of the most abundant biological resources, some microorganisms have adapted to habitat contaminated with toxic metals, and thus evolved powerful tactics for remediating polluted environment while recycling metal resources [7, 10], and some review articles on the biosynthesis of MNPs using diverse microorganisms including bacteria, yeast, fungi, alga, etc. and their applications have been published in recent years [1, 2, 6, 7, 10].

**Fig. 1:**
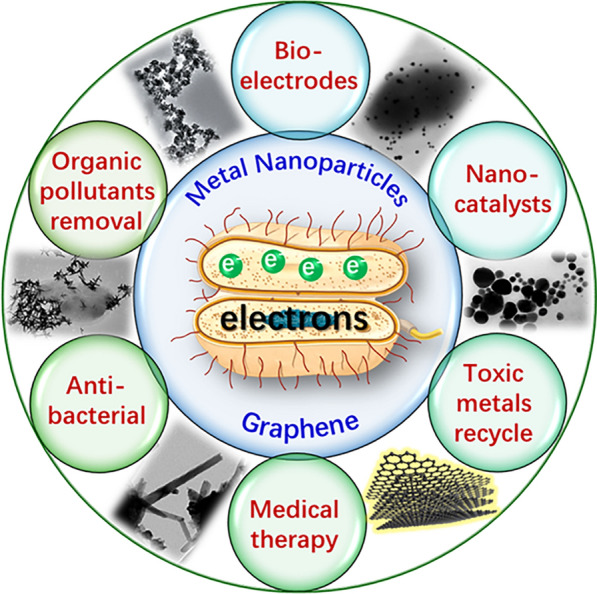
Overview for biogenic nanostructured materials (metal nanoparticles and graphene) and their diverse applications

Nevertheless, our particular concern is dissimilatory metal-reducing bacteria (DMRB) like *Shewanella* and *Geobacter* species that are capable of peculiar extracellular electron transfer (EET). Due to their unique functions on electron exchange with extracellular environments, DMRB have aroused intensive research enthusiasm over the past two decades, not only on uncovering their ecological distributions and functions in nature but also on developing a series of novel technological systems in many interdisciplinary areas such as biogeochemistry, bioelectrochemistry, environmental science & engineering, and nanobiotechnology [11–14]. In absence of electron acceptors that are available intracellularly (e.g. oxygen and soluble molecules with high oxidation states), these bacteria can also anaerobically oxidize organic matters inside cells, and then transfer electrons released across their cell envelope barriers to extracellular redox-active minerals (electron acceptors), such as those that contain iron (Fe^2+^ and/or Fe^3+^) and manganese (Mn^3+^ or Mn^4+^), to drive the biogeochemical cycling of elements [15–18]. They also can use solid electrodes like graphite as terminal electron acceptors, thereby coupling bacterial intracellular energy metabolism with bioelectricity production, and such a system is referred to as microbial fuel cell [13]. These bacteria possessing EET ability are generally termed as electroactive microorganisms, and *G. sulfurreducens* and *S. oneidensis* MR-1 are the two most important model strains [19]. More impressively, many strains of DMRB have functions on the biosynthesis and bioassembly of nano-sized materials associated with their versatile EET features, especially MNPs. With great advances in elucidating bacterial EET mechanisms over the last decade, many noble metal nanoparticles [20–24], their alloys [25, 26], metal oxides [27, 28] and chalcogenides [29, 30] have been synthesized by *Shewanella* and *Geobacter* species. Besides, the bacterial EET pathway that pumps electrons out of the cells enables the extracellular reduction of metal ions to form MNPs in the culture, which is beneficial to their subsequent separation and purification. The biogenic metal nanomaterials are promising in many application fields (Fig. 1).

*Shewanella* and *Geobacter* species are able to produce graphene through the biological reduction of graphene oxide (GO), a two-dimensional honeycomb-structured single atom layer carbon material with high hydrophilicity and biocompatibility. More interestingly, bacterial EET-driven biosynthesis provokes an interesting tactics of the self-assembling bio-abiotic hybrid composed of bacterial cell and inorganic nanomaterials, which exhibit many novel properties originated from their intimate interractions, leading to broader applications.[31–34] Moreover, with the innovation and development of various biotechnologies represented by synthetic biology, the controllable biosynthesis of nanomaterials with well-defined structures and features by virtue of rationally tailoring the EET pathway becomes feasible.

Taking into consideration of an ever-growing research enthusiasm and great achievement, a critical review focusing especially on the biosynthesis of nanomaterials inspired by bacterial EET is needed.

## Bacterial extracellular electron transfer

The earliest observation of microbial capacity to exchange electrons with extracellular environments was observed by Potter in the early 1900s [35], while the research upsurge started after the discoveries of two typical DMRB (*Shewanella* and *Geobacter* spp.) three decades ago [15, 17]. Since then, extensive studies have devoted to molecular mechanisms by which DMRB cells exchange electrons with extracellular redox-active substances, particularlly solid electrodes and inorganic minerals, as well as their functions on the earth's ecology and geochemical element cycle [11]. Meanwhile, a series of microbial electrochemical technologies, such as microbial fuel cell, microbial electrolysis cells, microbial desalination cell and microbial electrosynthesis, have also emerged on the basis of electron exchange between electroactive microorganisms and solid electrodes [13, 36].

**Fig. 2:**
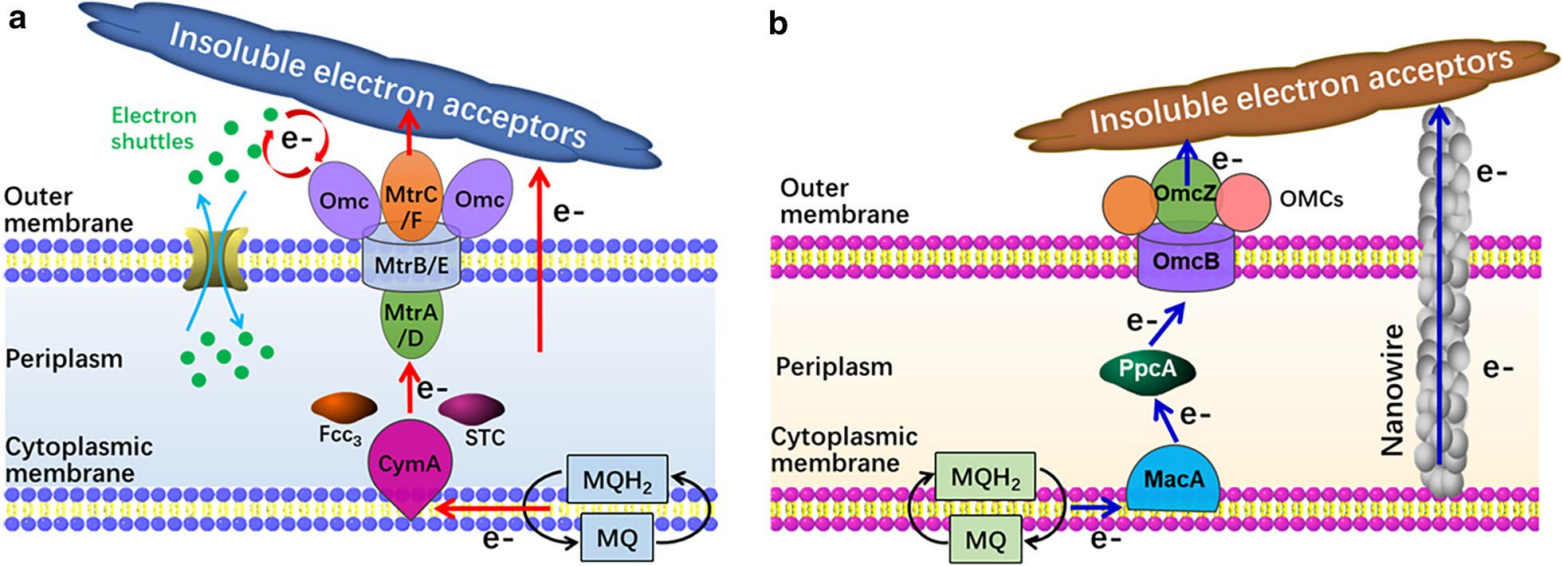
Mechanistic diagram for the bacterial EET. **a** *Shewanella oneidensis* MR-1, and **b** *Geobacter sulfurreducens*

As shown in Fig. 2, the bacterial EET process can carry out either directly or indirectly [11, 13, 36]. For the model strain of *S. oneidensis* MR-1, its direct EET relies on a metal-reducing (Mtr) conduit consisting of six multi-heme *c*-type cytochromes (*c*-Cyts): CymA, Fcc_3_, MtrA, MtrC, OmcA and small tetraheme cytochrome (STC), and a porin-like MtrB located on the outer-membrane, which work together for electron trans-membrane transport [11]. In detail, CymA oxidizes the menaquinol pool located in the cytoplasmic membrane wherein the electrons come from reducing equivalents produced during intracellular energy metabolism, and transfers electrons to the periplasmic redox proteins Fcc_3_ and STC. Proteins MtrA, MtrB and MtrC form a ternary complex across the outer-membrane responsible for transporting electrons from the periplasmic space to bacterial cell surface [37–40]. Then MtrC and OmcA can interact with each other and deliver electrons to extracellular electron acceptors (e.g. solid electrodes and insoluble minerals) directly contacted with bacterial surfaces [41–44]. Notably, the direct physical contact between extracellular electron acceptors and bacterial out-membrane *c*-Cyts (MtrC and OmcA) is necessary for this EET mode. The Mtr pathway of *S. oneidensis* MR-1 is the best-characterized EET route so far, and its homologues are found in all sequenced *Shewanella* species [11, 45]. In the case of indirect pattern, *S. oneidensis* MR-1 secretes small redox-active molecules such as flavins or other quinones to execute electron shuttling back and forth between cells and external electron acceptors [46, 47]. The indirect EET mode relies on the abilities of these endogenous electron shuttles to effectively pass through the cell membrane barrier. However, the two EET pathways seem to be not independent. For instance, out-membrane *c*-Cyts have been evidenced to serve as terminal reductases for the extracellular reduction of electron shuttles [45, 48]. Besides, some studies have demonstrated that the flavins can act as the co-factors for outer-membrane MtrC and OmcA to accelerate interfacial electron transfer rate [49, 50].

Multiheme *c*-Cyts, especially diverse types of Omc proteins, are also identified to play key roles in the EET process of *G. sulfurreducens*, and these *c*-Cyts work collectively to transfer electrons from the quinol pool existed in the cytoplasmic membrane, across the periplasm and outer membrane to the bacterial outside [11, 51, 52]. For example, the deletion of OmcZ (an out-membrane *c*-Cyts of *G. sulfurreducens*) resulted in alomost failure in EET capacity [53, 54]. Moreover, it should be pointed out that *G. sulfurreducens* can generate specific conductive pili that are referred to as bacterial nanowires with metal-like conductivity. The bio-nanowires serve as an alternative direct pathway to achieve more effective electron transport especially in biofilms [55, 56].

There are convincible evidences to indicate that extracellular polymeric substances (EPS), a complex biopolymer mixture produced by bacterial cells, are involved in the EET process [57]. For example, about 20 redox proteins including *c*-Cyts of MtrC and OmcA were detected in EPS from *Shewanella* sp. HRCR-1 biofilms [58]. Moreover, EPS matrices extracted from *S. oneidensis* MR-1 have been confirmed to be electrochemically active with the clear observation of redox peaks of *c*-Cyts by voltammetry measurement [59, 60]. On account of the existence of vast functional groups like carboxyl, phosphoric, amine and hydroxyl groups, the EPS matrices are expected to be relevant to the formation of MNPs because of their electrostatic affinity for metal ions.

For more details about mechanisms underlying EET, refer to some previous reviews [11–13, 19, 36].

## Biosynthesis of metal nanoparticles

The versatile and vigorous EET features of *Shewanella* and *Geobacter* species not only enable them to output electrons for extracellular reduction of redox-active minerals and electrodes, but also inspire a promising function on the bio-manufacturing of nanomaterials especially MNPs when using different metal ions as electron acceptors. Some inorganic nanoparticles are naturally presented in microorganisms such as magnetosome particles in magnetotactic bacteria [61]. The biological mineralization or/and reduction of hydrosoluble metal ions to form low-bioavailable solid-phase particles are inherent behaviors for many bacteria, by which they can get rid of the potential toxicity and stress effect caused by metal ions. In comparison to intracellular biosynthesis, bacteria possessing EET characteristic can generate MNPs outside cells or/and on the surface of cells via the reduction process driven by the transfer of extracellular electrons without the need of intracellular uptake of metal ions. Clearly the EET route endows the bacteria with a flexible prevention strategy to lower the risk of toxic metal ions, and thereby holds a promising prospect in biosynthesis, even the recovery of MNPs.

### Monometallic nanoparticle

**Fig. 3:**
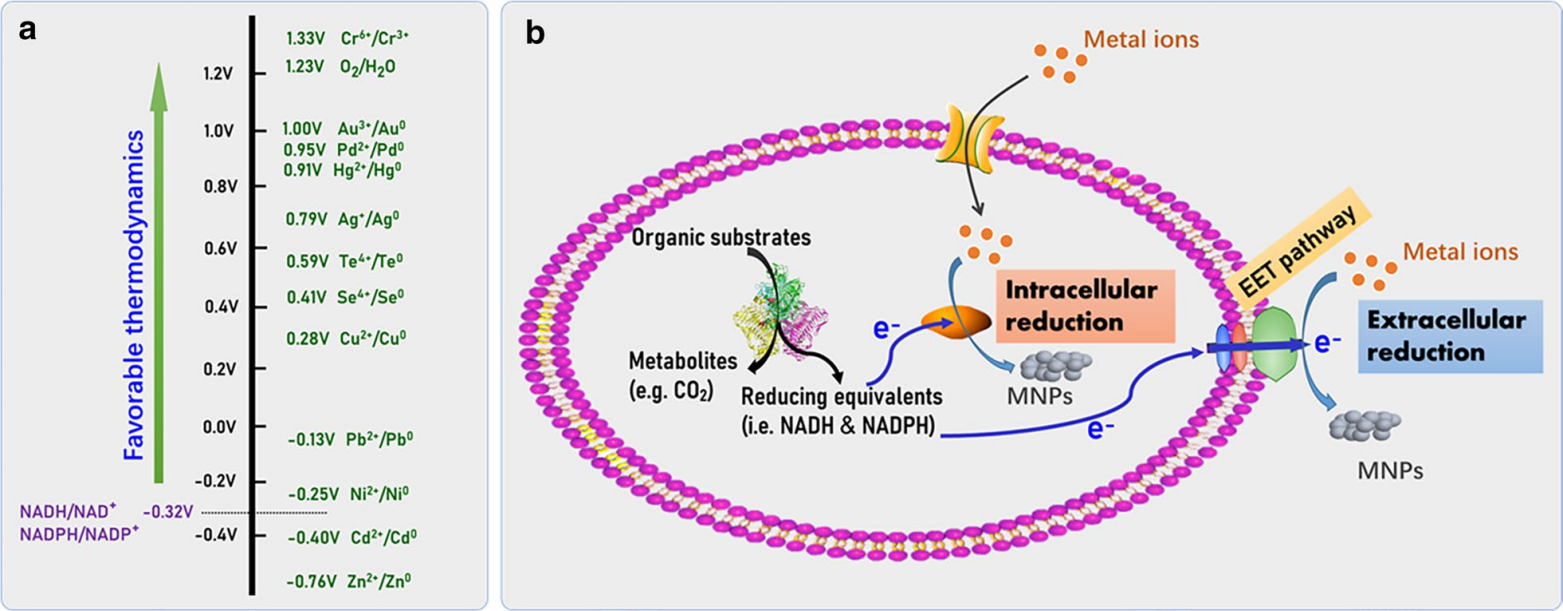
Illustration of the thermodynamic feasibility for microbial reduction of metal ions to elemental forms under anaerobic condition (**a**), and the biosynthesis of MNPs through microbial reduction of metal ions both inside and outside cells (**b**)

The intracellular reducing equivalents including NADH and NADPH produced by the oxidative metabolism of organic substrates are primary forces to drive the bioreduction reaction. Metal ions, including Au^3+^, Ag^+^, Pd^2+^, Se^4+^, Te^4+^ and Cu^2+^ with much higher redox potentials than NADH and NADPH (*E*^*0*^ ≈ − 0.32 V) [62], can be effectively reduced in theory to insoluble elemental metals inside cells as well as at extracellular matrix once the reducing forces are accessible through the EET pathways (Fig. 3). Noble metal nanoparticles are valuable materials with various applications such as in catalysis and medical sensing and diagnosis [63, 64]. The concept of green chemistry has spawned intensive research on the biosynthesis and recovery of noble MNPs [65]. On the other hand, other monometallic nanoparticles such as nanostructured chalcogen metalloids and copper nanoparticles have also been developed.

#### Gold nanoparticles

As a pioneering work, Suresh and co-workers reported the bio-fabrication of gold nanoparticles (Au-NPs) using *S. oneidensis* MR-1 cells as biological factories, which demonstrated the substantial ability of *S. oneidensis* MR-1 in the extracellular reduction of chloroaurate (Au^3+^), producing discrete spherical Au-NPs outside the cells with an average size of 12 ± 5 nm [20]. The biosynthesized Au-NPs were hydrophilic and not aggregated even after several months, and exhibited high biocompatibility with neither toxicity nor inhibition to both Gram-positive and Gram-negative bacteria, because they were likely capped by a detachable protein/peptide coat during the formation process. To reveal the contribution of the bacterial surface structure on Au-NPs production, Ishiki and co-workers tracked the bioformation process of Au-NPs on *S. oneidensis* MR-1 cell surface by using electron microscopy, zeta potential and spectrometry analyses [66]. The authors found that both the extracellular electron transport and the secretion of extracellular polysaccharide (EPS) executed the Au^3+^ reduction and controlled characteristics of the produced Au-NPs such as their particle size.

Interestingly, the mutant of *S. oneidensis* MR-1 lacking proteins MtrC and OmcA, two important outer-membrane *c*-Cyts for direct EET, still could synthesize Au-NPs on the bacterial cell surface. Moreover, the biosynthesized Au-NPs grown on the cell surface were found to repair the damaged EET chain of the mutant strain to a certain degree [67]. These findings signify the possible existence of alternative routes responsible for the reductive synthesis of Au-NPs in addition to the Mtr pathway. Likewise, the ability in biological synthesis of Au-NPs has been observed later for other *Shewanella* species including *S. putrefaciens* CN32 [68], *S. haliotis* [69] and *Shewanella* sp. CNZ-1 [70]. The size distributions of Au-NPs were dependent on various reaction conditions including bacterial biomass loading, concentrations of electron donors and gold ions, pH environment, etc.

In contrast to *Shewanella*, *Geobacter* species can construct a thick and dense biofilm matrix when grown on an electrode surface [71]. The well-reductive biofilm matrix with high availability of electrons is expected to reduce gold ions to Au-NPs, and biomolecules in the biofilm matrix can serve as active sites for the nucleation and stabilization of Au-NPs [72]. Inspiringly, Tanzil and co-workers made an attempt to synthesize Au-NPs using an electrode-respiring *G. sulfurreducens* biofilm, and Au-NPs with an average size of 20 nm were formed inside the extracellular matrix of the biofilm [24]. The in-situ bio-formation of Au-NPs was also realized in another *G. sulfurreducens*-electrode biofilm by the slow addition of NaAuCl_4_ precursors during its development [73]. These achievements demonstrates the potential tactics for constructing Au-NPs hybridized electroactive biofilms, and collaboration between inorganic nanoparticles and biomacromolecules is worth looking forward to.

#### Silver nanoparticles

Silver nanoparticles (Ag-NPs) have showed their applications in over 200 products including antimicrobial coatings, medical devices, molecular diagnostics, sensors, electronics and fillers [74], making biological methodologies for Ag-NPs synthesis attractive. The extracellular biosynthesis of silver-based single nanocrystallites of well-defined composition and homogeneous morphology has been observed when *S. oneidensis* MR-1 cells were inoculated in aqueous silver nitrate solution [21]. The produced biogenic Ag-NPs with monodispersed nanospheres ranging from 2 to 11 nm showed higher toxicity towards both Gram-positive (*Bacillus subtilis*) and Gram-negative (*Escherichia coli* and *S. oneidensis*) bacteria compared to those chemically synthesized counterparts. Specifically, the reduction of Ag^+^ to form Ag-NPs was also achieved by using the EPS extracted from *S. oneidensis*, and *c*-Cyts present in the EPS matrix contributed to the biological reduction [75]. However the EPS-enabled reduction process is usually compromised by the relatively low reducing rate, because the reducing capability of EPS lacking constant energy supply from bacterial cells is always weak. To address such a challenge, a light-induced tactic was developed, where the reduction of Ag^+^ by EPS from *S. oneidensis* MR-1 was accelerated significantly by illumination treatment under both visible and UV light [76]. Besides, the outer membrane *c*-Cyts of *S. oneidensis* MR-1 exhibited a significant effect on the size and activity of extracellularly synthesized Ag-NPs, and the lack of MtrC and OmcA reduced the particle size, but increased the antibacterial activity of the biogenic Ag-NPs [77].

#### Palladium nanoparticles

Palladium (Pd) is attracted ever-increasing interest in both scientific and industrial communities due to its similar high activity in various catalytic reactions but much higher earth reserves compared to platinum (Pt) [78].

**Fig. 4:**
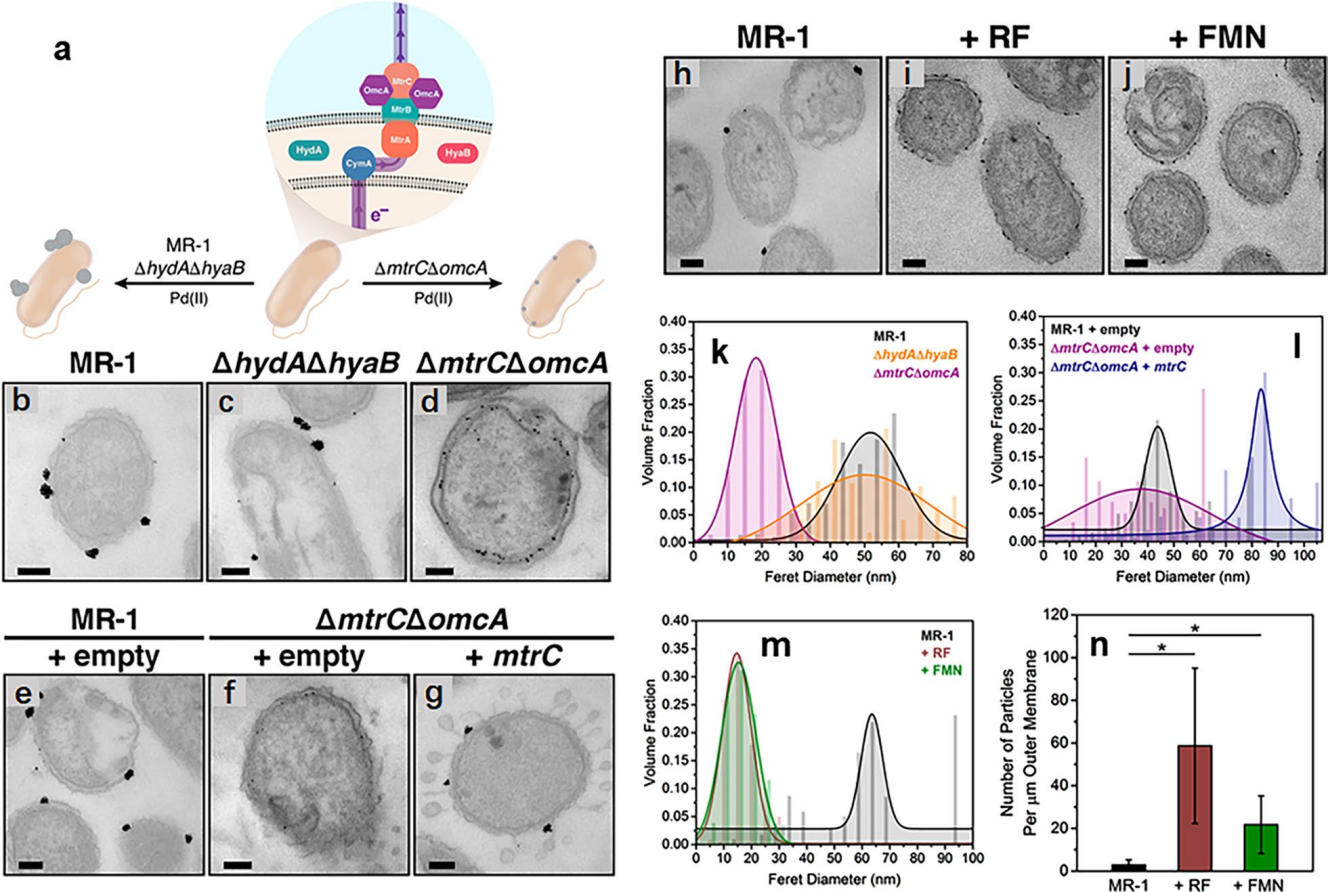
Functions of EET components (MtrC and electron shuttle flavins) on Pd-NPs formation by *S. oneidensis* MR-1. **a** General diagram of EET pathway and genotypic effects on Pd-NPs formation. Thin section transmission electron micrographs of different strains: **b**, **h** MR-1, **c** *ΔhydAΔhyaB,* **d** *ΔmtrCΔomcA*, **e** MR-1 with an empty vector, *ΔmtrCΔomcA* with an empty vector **f** or *mtrC* vector **g**, and MR-1 with **i** 100 μM riboflavin (RF) or **j** 100 μM flavin mononucleotide (FMN) after 2 h reactions with 100 μM Pd^2+^. Scale bars represent 100 nm. **k**–**m** Nanoparticle size distributions were determined by analyzing thin section transmission electron micrographs for each strain. **n** Outer membrane-normalized particle counts for un-supplemented and flavin-supplemented MR-1 [81]

*Shewanella* and *Geobacter* species have been widely used to synthesize Pd-NPs due to their dissimilatory reduction properties. Besides, they also provide an alternative approach to recycle Pd resources from wastewater. *S. oneidensis* MR-1 can reduce soluble Pd^2+^, and thus precipitate Pd-NPs either on the cell wall or inside the periplasmic space in the presence of electron donors such as hydrogen gas, formate, lactate, pyruvate and ethanol [79]. Moreover, the size distribution and catalytic reactivity of Pd-NPs produced by *S. oneidensis* MR-1 could be tailored by changing electron donors or controlling the ratio of precursor ions to the bacterial cells [80]. The functions of the EET components of *S. oneidensis* MR-1, including outer-membrane MtrC and electron shuttles such as flavins, on the Pd-NPs biosynthesis were elaborated recently by Dundas and co-workers, and with the variation of the EET components, observable changes in the rate of their biosynthesis, size distribution and cellular localization were observed (Fig. 4) [81]. They also found that MtrC was a critical machine for delivering electrons to Pd^2+^ and mediating Pd-NPs nucleation, and the particle size decreased in a dose-dependent manner with the increase in flavin concentration, but the particle number per cell increased. These findings provide substantial evidences for the concept that bacterial EET coordinates the biological formation of inorganic nanoparticles. Given their genetic tractability, *Shewanella* spp. are expected to be developed as a model platform for tracing nanoparticle biogenesis and engineering functional nanoparticles for emerging applications.

The ability of *G. sulfurreducens* PCA to reduce Pd^2+^ using acetate as electron donors at neutral pH and physiological temperature was firstly documented by Pat-Espadas and co-workers [82]. The authors found that the productivity of Pd-NPs was greatly enhanced after the addition of redox mediator (anthraquinone-2,6-disulfonate, AQDS). In addition to the Pd-NPs on the bacterial surface, others detached from the cells were also observed, probably owing to that the Pd^2+^ around the bacterial cells accepted electrons by AQDS mediators rather than directly from membrane-bound *c*-Cyts [83]. The production of Pd-NPs outside the cells has also been demonstrated in another research with *G. sulfurreducens* PCA [23]. Given that these extracellular nanoparticles can be easily extracted from bacterial cultures through simple centrifugation, the extracellular biosynthesis is greatly beneficial to industrial production. Very recently, the global transcriptional analysis of *G. sulfurreducens* PCA revealed the crucial role of electrically conductive pili in Pd^2+^ reduction [84], but more investigations are needed to elaborate the underlying mechanism. Meanwhile, the expression levels of *c*-Cyts during Pd^2+^ reduction were found to be distinctly different to that when either Fe^3+^ or electrode was used as electron acceptors [84], which further implies the complicated and changeable mechanism underlying the bacterial extracellular reduction process.

#### Nanostructured chalcogen metalloids

Elemental selenium (Se) and tellurium (Te) that belong to chalcogen metalloids with unique *p*-type semiconductor characteristics have applications in electronic, photoelectronic and optic products, glasses, pigments and sensors [85]. Both of them have a wide range of chemical valence states from − 2 to + 4, and exist in several different forms, including soluble oxyanions (SeO_4_^2−^, SeO_3_^2−^, TeO_4_^2−^, TeO_3_^2−^), insoluble elemental states (Se^0^, Te^0^) and inorganic chalcogenides (Se^2−^, Te^2−^), depending on prevailing redox conditions. Bacteria play a key role in their biogeochemical cycles [85]. As early as in 2005, *S. oneidensis* MR-1 was reported to reduce selenite (SeO_3_^2−^) and tellurite (TeO_3_^2−^) under anaerobic conditions to produce nanosized Se^0^ and Te^0^ deposits [86]. Since then, many *Shewanella* strains have been documented with biosynthetic abilities of zero-valent Se nanoparticles (Se-NPs) and Te nanoparticles (Te-NPs) from oxyanions [87–97].

The periplasmic fumarate reductase FccA of *S. oneidensis* MR-1 has been identified to reduce selenite during the bio-formation of zero-valent Se-NPs [87]. Impressively, the selenite reduction process in *S. oneidensis* MR-1 cells could be well tuned by electron shuttles such as AQDS and riboflavin. The addition of these electron shuttles not only accelerated markedly the selenite reduction rate but also diverted the location of Se-NPs from inside to outside cells [88]. Moreover, the shape, size distribution and formation rate of Se-NPs could be effectively tuned by multiple biosynthetic reaction conditions, including bacterial biomass, selenite concentration, dissolved oxygen, incubation temperature and reaction time [89, 97]. For example, *Shewanella* sp. HN-41 produced well-shaped spherical Se-NPs under either anaerobic or hypoxic conditions, while irregular-shaped products with smaller sizes were developed under oxygen-saturated conditions [89]. The biomass density of *Shewanella* sp. HN-41 and selenite concentration also impacted the selenite reduction rate and the particle size distribution and productivity of Se-NPs [97]. More impressively, the spherical Se-NPs produced by *Shewanella* sp. HN-41 were found to be rapidly transformed into nanowires (1-D structure) or/and nanoribbons (2-D structure) after being transferred from the aqueous phase to the dimethyl sulfoxide (DMSO) solution [90]. The crystallinities and shapes of Se-NPs depended on the DMSO concentration. These findings hint a possible solution for the controllable production of Se-NPs with shape-dependent functions.

*S. oneidensis* MR-1 could also reduce tellurite to elemental tellurium under an anaerobic condition, thereby leading to the intracellular accumulation of needle-shaped crystalline Te nanorods [93]. Interestingly, the tellurite reduction rate of *S. oneidensis* MR-1was promoted distinctly in the presence of Fe^3+^ acting as a co-existing electron acceptor [94]. More surprisingly, only extracellular crystalline Te nanorods with the length of 240 nm and width of 25 nm were observed after the addition of Fe^3+^. The extracellular formation of tellurite complex precipitates triggered by the biological production of Fe^2+^ from Fe^3+^ under the anaerobic condition was suggested to be responsible for the exclusive formation of the extracellular products, through which these generated precipitates were reduced into Te nanorods by *S. oneidensis* MR-1 extracellular respiration [94, 98]. Except for *S. oneidensis* MR-1, the strains of *S. baltica* GUSDZ9[96] and *Shewanella* sp. Taa[91] were also documented to produce Te nanorods and globular particles, respectively. Therefore, *Shewanella* species can achieve dual merits of detoxifying harmful tellurite and synchronously recycling industry-applicable Te resources.

Biosynthesis of Se- and Te-NPs has also been documented with *G. sulfurreducens*.[95, 99, 100] *G. sulfurreducens* has been proved to reduce Se^4+^ to produce Se nanospheres using both acetate and hydrogen as electron donors, and the reduction rate depended on the used electron donors as well as whether or not addition of redox mediators, but a fraction of Se elements was reduced further to Se^2−^ because of the instability of Se^0^.[95] Jahan et al. found that a porin-like protein ExtI from *G. sulfurreducens* participated the reduction of selenite and tellurite because the *extI* deficiency caused an obvious decrease in the reduction ability [99]. Therefore, ExtI was hypothesized to play a role not only in selenite uptake but also in Se-NPs formation/excretion [100]. Nevertheless, the bio-formation mechanism of Se- and Te-NPs is still unclear, and further in-depth research is needed.

#### Copper nanoparticles

Given its high natural abundance and relatively low cost, the applications of copper (Cu) and Cu-based NPs in catalysis and antibacterial products have always generated interest [101, 102]. *S. oneidensis* MR-1 can directly reduce Cu^2+^ to its elemental form in the absence of oxygen. In a study case, nanostructured Cu precipitates (20–50 nm) were formed inside the cells when *S. oneidensis* MR-1 were incubated anaerobically in 0.05 mM CuSO_4_ solution with the addition of lactate as the electron donor [103]. Meanwhile, there were also a small amount of large-sized aggregates over 200 nm observed in the extracellular matrix. However, a research on biosynthesis of Cu-NPs using *S. loihica* PV-4 exhibited inconsistent results, where there were abundant small-sized Cu-NPs (10–16 nm) on the bacterial cell surface while a little bit of intracellular precipitates [104]. Very recently, high-crystallizd Cu-NPs with a small diameter ranging from 4 to 10 nm were grafted on carbon nanotube (CNT) surfaces through a biosynthetic approach using *S. oneidensis* MR-1, which further proves the ability of *Shewanella* spp. in extracellular Cu^2+^ reduction [105].

### Bimetallic nanoparticles

Integration of two kinds of metals into single bimetallic nanoparticles often leads to more unique and superior physicochemical properties than their monometallic counterparts [106]. Synthesis of bimetallic nanoparticles is therefore receiving attention. For instance, bimetallic Pd-Au nanoparticles have been applied as excellent catalysts for different reactions, including but not limited to carbon dioxide reduction [107], oxygen reduction reaction [108], methanol oxidation [109], nitrite reduction [110], and selective detection of reactive oxygen and nitrogen species [111]. Great effort has been placed on developing physical and chemical methodologies capable of synthesizing bimetallic nanocrystals with well-defined structures and rational-tuned features [106]. Nevertheless, how to fabricate functional bimetallic nanoparticles by a more facile and green approach encourages researchers to develop an alternative protocol from microbial perspective [112].

As what elaborated before, various kind of monometallic nanoparticles especially noble metals can be biosynthesized easily by bacteria possessing the EET ability. Accordingly, *S. oneidensis* MR-1 has been inspired to biosynthesize Pd-NPs and Au-NPs on bacterial cell surfaces. The OmcA/MtrC complex was proved experimentally to work as a key machine in the bioreduction of Pd^2+^ and Au^3+^, indicating the crucial role of the direct electron transfer endowed by out-membrane *c*-Cyts in the biosynthetic process of noble nanoparticles, and intriguingly, the biosynthesized Pd-NPs and Au-NPs that were separated from each other could fuse together after a facile hydrothermal reaction, thereby becoming Pd/Au alloys with outstanding electrocatalytic activity [25]. This work presents a feasible case of green biosynthesis of functional bimetallic nanoparticles. More impressively, *S. oneidensis* MR-1 was found recently to be capable of directly producing biogenic Pd/Pt-NPs when incubated in the solution of Pd^2+^ and Pt^4+^ at two independent laboratories [26, 113]. The produced bimetallic Pd/Pt-NPs exhibited much superior catalytic activity towards organic pollutants (e.g. 4-nitrophenol) compared to the monometallic counterparts (Pd-NPs and Pt-NPs). Additionaly, electron shuttles such as AQDS were demonstrated to not only promote the reduction efficiency of Pd^2+^ or/and Pt^4+^ but also lower the sizes of both mono- and bi-metallic nanoparticles [26]. Although only a few of literatures about the biosynthesis of bimetallic nanoparticles, we believe that much more research activities in this direction are look forward to in the future because of their extraordinary characteristics and promising applications.

### Magnetite nanoparticles

Some DMRB can respire a broad range of high oxidation state minerals such as Fe^3+^-oxyhydroxides, so as to take part in their biogeochemical cycles [11, 114]. Such a natural biotransformation process offers a practical solution to the production of functional mineral-derived nanomaterials. Magnetite (Fe_3_O_4_) nanoparticles have been particularly highlighted in terms of their fruitful applications in cancer therapy, drug delivery, chemical and biological sensors, magnetic catalysts, magnetic data storage and environmental remediation, due to their specific magnetic properties and high surface reactivities in nanoscales [115–118].

Magnetotactic bacteria naturally produce intracellular magnetite nanoparticles (magnetosomes) with high purity and crystallinity, uniform morphology and grain-size distribution [61]. In contrast to the intracellular biomineralization of magnetotactic bacteria, the DMRB tend to produce extracellular magnetite nanoparticles by the dissimilatory reduction approach outside cells, which can bring great convenience to product recovery. Many iron-reducing bacteria including *S. oneidensis* MR-1 [119, 120], *Shewanella* sp. HN-41 [121, 122], *S. piezotolerans* WP3[123] and *G. sulfurreducens*[124–132] have been exploited to produce well-defined magnetite nanoparticles through the dissimilatory reduction of poorly crystalline Fe^3+^-oxyhydroxides. Besides, the biosynthesized magnetite nanoparticles could well support nanostructured Pd for significent improvement on functionality and applicability [125, 133].

What should be noted is that, the biosynthetic processes are tunable and scalable with respect to particle size, surface reactivity, and magnetic, optical and thermal properties. The types of bacteria and organic substrates as electron donors, as well as the density of bacterial biomass, mineral precursors and redox mediators have been demonstrated to be of great significance to the products [119, 120, 122, 126]. It is well known that hetero-atom doping can tune the structure and property of nanomaterials. The biological incorporation of Co^2+^ and Zn^2+^ into the structure of magnetite nanoparticles has been realized using *G. sulfurreducens*, and the produced Co and Zn-doped nanoparticles showed enhanced magnetic properties dependent on the hetero-atom doping level [127, 128, 132]. Although the production of biomagnetite nanoparticles by iron-reducing bacteria has yet to be taken up in industry, an attempt on scale-up production from laboratory- to pilot plant- scale has been carried out [129]. In this trial, no significant impact was observed on the nanoparticle size, magnetic property and surface reactivity of the products during the scale-up from 10 mL to 10 L. Besides, the formation of other iron mineral nanomaterials such as siderite (FeCO_3_) nanoparticles and goethite (*α*-FeOOH) nanowires has been also observed in iron-reducing bacterial cultures containing Fe^3+^-oxyhydroxides [27, 122].

### Metal chalcogenide nanoparticles

Metal chalcogenides are emerging as promising nanomaterials due to their unique size- and shape-tunable optoelectronic, physicochemical and biological properties. Considerable interest on innovations of methodologies for controllable synthesis of them has been raised among academic and scientific communities. The biological synthetic route with benefits of low energy consumption and less impact on environment is beyond all doubt highly preferred nowadays [134, 135].

*Shewanella* spp. are capable of using thiosulfate, tetrathionate, sulfite and elemental sulfur as terminal electron acceptors for anaerobic respiration, and consequently produce S^2−^ that has high affinity towards a variety of metal ions to form metal sulphides [136–139]. Lee et al. reported a pioneering work on the biosynthesis of an extracellular network of filamentous arsenic-sulfide (As-S) nanotubes (20–100 nm in width and up to 30 μm in length) by *Shewanella* sp. HN-41 under anaerobic conditions, in which the transformation process was triggered by the biological reduction of As^5+^ and thiosulfate [29]. The produced yellow As-S filamentous nanotubes behaved as both electrical conductivity of metals and photoconductive characteristics of semiconductors, indicating their promising applications in nano- and opto-electronic devices. Whereafter, various ternary and quaternary chalcogenides including As-S-Se, As-Cd-S and As-Cd-S-Se nanotubes were synthesized using the above-mentioned As-S nanotubes as templates through microbiological or/and abiotic modifications under ambient conditions [140]. Impressively, the biosynthesized As_4_S_4_ has been successfully adopted as Li-ion active electrode materials after its low conductivity was effectively improved through the introduction of high-conductive graphene [141]. Strain of *Shewanella* sp. ANA-3 was reported to rapidly synthesize extracellular As_2_S_3_ nanofibers with a high yield (504 mg per liter of the culture, 82% of the maximum theoretical value) through anaerobical reduction of As^5+^ and thiosulfate [142].

It has been widely demonstrated that the biological production of iron sulfides can occur as a consequence of dissimilatory reduction of diverse ferric iron (Fe^3+^) by *Shewanella* spp., including both dissolved ions and insoluble minerals when they coexist with thiosulfate, sulfite or/and elemental sulfur [143–148]. To our best knowledge, *S. oneidensis* MR-1 [144, 147, 148], *S. loihica* PV-4[143] and *S. putrefaciens* CN32[146] have been documented capable of biosynthesizing nanostructured FeS under anaerobic conditions. The structure, size and reactive activity of these biogenic FeS are, to a great extent, dependent on the synthesis conditions. Very recently, Yu et al. investigated the effect of biogenic S^2−^ release rate that could be controlled by the initial thiosulfate dosage on the particle size distribution of FeS-NPs, and found that the Fe^3+^ was mainly reduced by the *S. oneidensis* MR-1-produced S^2−^ rather than the cells themselves once the initial thiosulfate concentration exceeded 5 mM [148]. More importantly, the authors also revealed that the biogenic S^2−^ release rate not only altered the Fe^3+^ reduction manner but also tuned the particle size of FeS products that showed a clear tendency to increase with increasing the biogenic S^2−^ release rate. This work shows a new strategy to realize the controllable synthesis of biogenic nanoparticles. In another interesting work, Fe^2+^ released from the metal-complex dye naphthol green B through biodegradation by *S. oneidensis* MR-1 was used to produce FeS-NPs after reacting with biogenic S^2−^, suggesting an environmental-friendly way for effectively coupling pollutant degradation and nanomaterial biosynthesis [149].

**Fig. 5:**
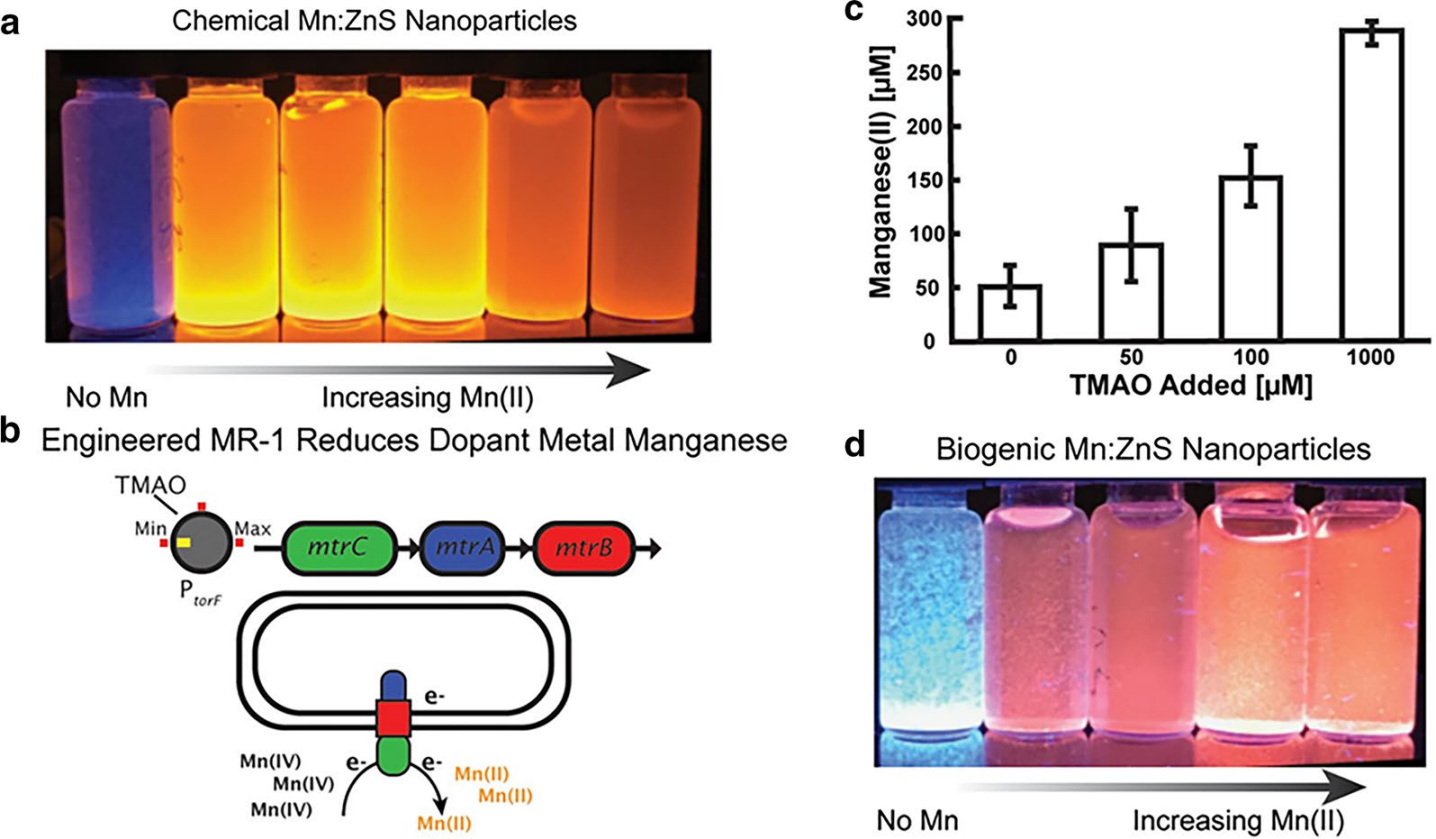
Controlling manganese doping of ZnS quantum dots by engineered *S. oneidensis* JG3631 with the inducible expression of MtrCAB complex. **a** Chemically synthesized Mn doped ZnS NPs with varying optical properties, **b** the amount of the inducer (TMAO) regulating expression of MtrCAB complex that performs extracellular reduction, **c** relationship between Mn^2+^ concentration and the addition of TMAO, **d** biogenic Mn doped ZnS quantum dots with varying optical properties as a function of Mn^2+^ concentration [155].

The biogenic S^2−^ produced by *S. oneidensis* MR-1 has also been applied to synthesize Ag_2_S nanospheres with a mean diameter of 9 ± 3.5 nm [150]. The produced biogenic Ag_2_S-NPs showed high biocompatibility without inhibition and cytotoxicity to both prokaryotic bacteria and eukaryotic cell lines, which was primarily attributed to the formed protein/peptide coating on the surfaces of biogenic nanoparticles during the biotransformation process [150, 151]. Small-sized and homogenous-shaped CuS-NPs produced by *S. oneidensis* MR-1 were reported as a candidate for photothermal therapy, which displayed a high photothermal conversion efficiency of 27.2% because of their strong absorption under infrared light [152]. Xiao et al. discovered a complex hollow CuS nano/micro shells self-assembled on the *S. oneidensis* MR-1 cell surface, which possessed a hierarchical structure leading to a significant enhancement of Cr^6+^ removal capacity [153]. Spherical ZnS nanocrystals with an average diameter of 5 nm were bio-fabricated through utilizing biogenic S^2−^ produced by *S. oneidensis* MR-1 to precipitate Zn^2+^, and the product showed a high level of photodegradation efficiency of rhodamine B [154]. Recently, Chellamuthu et al. developed a genetic-control strategy to biosynthesize manganese-doped ZnS (Mn:ZnS) nanoparticles with different doping levels (Fig. 5) [155]. The authors found that the Mn doping level changed as a function of added inducer when the engineered strain of *S. oneidensis* with inducible expression of MtrCAB complex was used to control the reduction of Mn^4+^ oxide. Impressively, these biogenically produced Mn:ZnS-NPs showed comparable physical and optoelectronic properties to chemically synthesized quantum dots. This work illustrates the promise of implementing synthetic gene circuits to controllable tune biogenic nanoparticles.

Cadmium chalcogenides, especially CdS, CdSe and CdTe, are considered as quantum dots when their physical size is lower than the exciton Bohr radii, and their unique size-dependent characteristics including broad UV excitation, narrow emission, bright photoluminescence and high photostability, endowing them cutting-edge applications in bioimaging, optical devices like light-emitting diodes, solar energy conversion and sensors [156, 157]. The biosynthesis of Cd-based quantum dots is flourishing due to its prominent economic and environmental benefits [157]. Both *S. oneidensis* MR-1[158, 159] and *G. sulfurreducens* PCA[30] have been adopted to bio-fabricate CdS-NPs under anaerobic conditions. To avoid the particle agglomeration, ionic liquids were introduced as soft templates to control crystal growth and assist assembly of biogenic CdS [158]. Notably, the in-situ synthesis of CdS quantum dots on bacterial cell surfaces can construct a biotic-abiotic hybrid system for efficient bio-photoelectric reductive degradation of some organic pollutants such as trypan blue and methyl orange [30, 159].

**Fig. 6:**
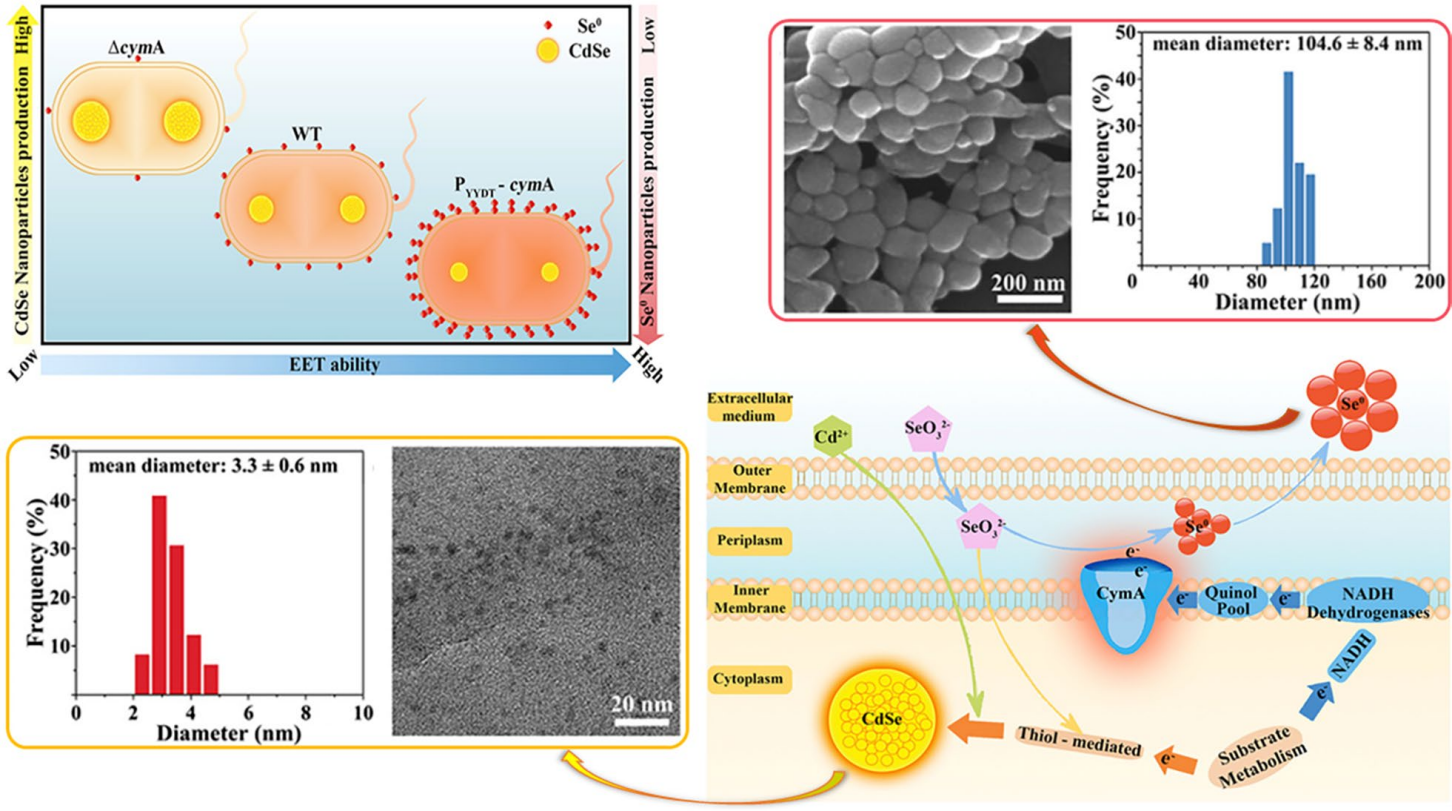
Schematic diagram for the EET-dependent synthesis of Se-containing nanoparticles by *S. oneidensis* MR-1 [160]

There is no doubt that how to realize high productivity and precisely control crystalline composition and structure always is a subject worth probing into. Tian et al. achieved the controllable production of Se-NPs and CdSe-NPs in *S. oneidensis* MR-1 cells, with fine-tuned composition and subcellular location, by genetically manipulating the EET chain (Fig. 6) [160]. The authors found that CdSe-NPs were mainly formed in the cytoplasm of the wild-type cells with Se^0^ nanoparticles in their periplasm. However, ultrafine, uniform-sized and fluorescence-characterized CdSe nanoparticles with an average diameter of 3.3 ± 0.6 nm were produced after the CymA-encoding gene was deleted, but much larger aggregates consisting of Se-NPs were abundantly generated when the CymA expression level increased [160]. It is quite clear that these findings fundamentally reconfirm the feasibility of EET regulation strategies for developing fine-controllable nanoparticles biosynthesis technologies [81].

For comparison, a series of MNPs biosynthesized by taking advantage of vigorous EET features of *Shewanella* and *Geobacter* species are listed in Table 1, along with their properties and particular applications.

**Table 1:** Summary of diverse MNPs biosynthesized by *Shewanella* and *Geobacter* species owning characteristic EET routes

MNPs	Morphologies, Sizes and dispersity	Bacterial strains	Synthetic sites	Properties and Applications	Refs.
Monometallic					
Au	Spherical NPs (~ 12 ± 5 nm), monodisperse and hydrophilic	*S. oneidensis* MR-1	Extracellular	Neither toxic nor inhibitory effect on Gram-negative and Gram-positive bacteria, high biocompatibility	[20]
	Spherical NPs (~ 10 nm)	*S. oneidensis* MR-1	Cell surface	–	[66]
	NPs (10–25 nm)	*S. oneidensis* MR-1	Cell surface and extracellular matrix	Partial repair of the damaged EET chain in *S. oneidensis* MR-1 mutant	[67]
	Spherical NPs (3–11 nm)	*S. putrefaciens* CN32	Both intra- and extra cellular	–	[68]
	Spherical NPs (10–30 nm)	*S. haliotis* (CTCC NO: M 2012444)	Extracellular	Size- and shape- dependent catalytic activity towards *p*-nitrophenol reduction	[69]
	Spherical NPs (~ 15 nm)	*Shewanella* sp. CNZ-1	Cell surface	Catalytic activity towards 4-nitrophenol reduction	[70]
	Spherical NPs (~ 20 nm)	*G. sulfurreducens* biofilm	Biofilm matrix	–	[24]
	NPs (5–50 nm)	*G. sulfurreducens* biofilm	Cell surface and Biofilm matrix	In situ promotion of EET and electricity production	[73]
Ag	Spherical NPs (2–11 nm, average 4 ± 1.5 nm), monodisperse	*S. oneidensis* MR-1	Extracellular	Bactericidal effect on Gram-negative and Gram-positive bacteria	[21]
	NPs (5–35 nm)	*S. oneidensis* MR-1 (EPS)	Extracellular	–	[75]
	Spherical NPs (8–10 nm)	*S. oneidensis* MR-1 (EPS)	Extracellular	–	[76]
	NPs (40.9 nm for wild-type strain, 24.4 nm for*△mtrC-omcA* strain)	*S. oneidensis* MR-1	Extracellular	Size-dependent antibacterial effect	[77]
	NPs (< 10 nm) grown on RGO	*S. oneidensis* MR-1	Extracellular	Catalytic degradation of 4-nitrophenol	[164]
Pd	Nonuniform NPs	*S. oneidensis* MR-1	Cell surface	Catalytic degradation of polychlorinated biphenyl or perchlorate	[79, 80]
	NPs (either < 10 nm or ~ 50 nm)	*S. oneidensis* MR-1	Periplasm or cell surface	–	[81]
	NPs (25.8 ± 7.8 nm)	*S. oneidensis* MR-1	Cellular or outer-membrane	Size- and distribution-dependent catalytic degradations of nitrobenzene and *p*-chlorophenol	[165]
	Nonuniform NPs ( 5–25 nm)	*S. oneidensis* MR-1	-	Catalytic reduction of 4-nitrophenol to 4-aminophenol	[22]
	NPs (~ 13 nm)	*S. oneidensis* MR-1	Periplasm or cell surface	High catalytic activity towards oxygen reduction reaction	[166]
	NPs (4–10 nm) without aggregation	*S. loihica* PV-4	Cell surface	High catalytic efficiency of Cr^6+^ reduction	[163]
	NPs (6–8 nm) loaded on TiO_2_ nanotubes	*S. oneidensis* MR-1	Extracellular	Photocatalytic degradation of methylene blue	[167]
	Nonuniform NPs	*G. sulfurreducens* PCA	predominantly in the EPS matrix surrounding cells	–	[23]
	NPs (5–15 nm)	*G. sulfurreducens* PCA	Cell surface or extracellular matrix when addition of AQDS	–	[82]
	NPs	*G. sulfurreducens* (DSM 12127)	On cell surface and inside the periplasm	Reduction of Cr^6+^ to Cr^3+^	[162]
Se	Spherical NPs	*S. oneidensis* MR-1	Either in medium or attached to cell surface	–	[86]
	Spherical NPs (~ 100 nm intracellularly, ~ 20 nm extracellularly)	*S. oneidensis* MR-1	Periplasmic space or cell surface	–	[87]
	NPs (∼50 nm)	*S. oneidensis* MR-1	Extracellular matrix	–	[88]
	Spherical NPs	*S. oneidensis* MR-1	Cell surface and extracellular matrix	–	[95]
	Spherical NPs (181 ± 40 nm, 164 ± 24 nm)	*Shewanella* sp. HN-41	Extracellular	–	[89]
	Nanowires and nanoribbons	*Shewanella* sp. HN-41	Extracellular	–	[90]
	Spherical NPs (100–400 nm)	*Shewanella* sp. 9a	Both outside and inside the cells	–	[91]
	Spherical NPs (50–100 nm)	*G. sulfurreducens*	Extracellular		[95]
	NPs (251–350 nm for wild-type strain, < 150 nm for *△extI* strain)	*G. sulfurreducens*	Extracellular		[100]
Te	Needle-like NPs	*S. oneidensis* MR-1	Either in cytoplasm or near cytoplasmic membrane	–	[86]
	Needle-shaped nanorods (length of 100–200 nm, width of ~ 10 nm)	*S. oneidensis* MR-1	Periplasmic and/or cytoplasmic spaces	–	[93]
	Needle-shaped nanorods (length of 89–240 nm, width of 7.5–25 nm)	*S. oneidensis* MR-1	Extracellular	–	[94]
	Spherical NPs under microaerobic conditions while nanorods under aerobic conditions	*Shewanella* sp. Taa	Both outside and inside the cells	–	[91]
	Nanorods (diameter of 8–75 nm)	*S. baltica* GUSDZ9 (Accession number: MF350629)	Intracellular	90% degradation of methylene blue dye and anti-biofilm activity against Gram-positive and Gram-negative human pathogens	[96]
Cu	NPs (20–50 nm)	*S. oneidensis* MR-1	Predominantly intracellular	Catalyzing azide-alkyne cycloaddition (an archetypal “click chemistry” reaction)	[103]
	NPs (10–16 nm) on CNT surfaces	*S. oneidensis* MR-1	Extracellular	Catalytic reduction of 4-nitrophenol to 4-aminophenol	[105]
	NPs (10–16 nm) with polycrystalline nature and face centered cubic lattice	*S. loihica* PV-4	Both on cell surface and inside cells	High antibacterial against *Escherichia coli*	[104]
Bimetallic					
Pd/Au	Alloy NPs (6.61 nm)	*S. oneidensis* MR-1	On cell surface	High electrocatalytic activity and durability for ethanol and formic acid oxidation	[25]
Pd/Pt	Small NPs (4.41 nm), flower-shaped NPs (59.90 nm)	*S. oneidensis* MR-1	On cell surface	Catalytic reduction of 4-nitrophenol (activity: bio-PdPt > Bio-Pd > Bio-Pt)	[26]
	Alloy NPs (3–40 nm), polycrystalline and face-centered-cubic structure	*S. oneidensis* MR-1	Inside and outside the cells	High-efficient catalytic reduction of nitrophenol and azo dyes	[113]
Pd/Ag	NPs on RGO	*S. oneidensis* MR-1	Extracellular	Catalytic reduction of 4- nitrophenol	[168]
Magnetite					
Magnetite (Fe_3_O_4_)	Spherical NPs (8–15 nm)	*S. oneidensis*	Extracellular	–	[120]
	Spherical NPs (26.7–37.7 nm, average 28.8 ± 3.4 nm)	*Shewanella* sp. HN-41	Extracellular	–	[121]
	Spherical NPs (4–6 nm)	*S. piezotolerans* WP3	Extracellular	–	[123]
	NPs (20–30 nm)	*G. sulfurreducens*	Extracellular	Ferrimagnetic carrier supporting Pd-NPs for the Heck reaction coupling iodobenzene to ethyl acrylate or styrene	[125]
	NPs (10–15 nm)	*G. sulfurreducens*	Extracellular	Reduction of Cr^6+^	[129]
Co-doped magnetite (CoFe_2_O_4_)	Nanocrystals containing 23 atom% Co (16–8 nm)	*G. sulfurreducens*	Extracellular	An improved magnetic property	[132]
Zn-doped magnetite (Zn_x_Fe_3-x_O_4_)	Spherical NPs (Zn-doping level dependent size)	*G. sulfurreducens*	Extracellular	An improved magnetic property	[127]
Metal chalcogenides					
As_x_S_y_	Filamentous nanotubes (diameter of 20–100 nm, lengths up to ~ 30 μm)	*Shewanella* sp. HN-41	Extracellular	Semiconductive and photoconductive	[29]
As_2_S_3_	Nanofibers (diameter of 20–600 nm, length up to 150 μm)	*Shewanella* sp. ANA-3	Extracellular	–	[142]
FeS	Nanosized colloids	*S. loihica* PV-4	Extracellular	Increased bioelectricity production	[143]
	Nanowire clusters	*S. oneidensis* MR-1	Extracellular	Long-distance EET	[144]
	Mackinawite	*S. oneidensis* MR-1	Extracellular	Accelerated dechlorination of trichloroethylene	[147]
	NPs (30–90 nm)	*S. oneidensis* MR-1	Extracellular	Removal of aqueous Cr^6+^	[148]
	NPs (~ 30 nm)	*S. oneidensis* MR-1	Both extracellular and intracellular	FeS-NPs biosynthesis coupling with naphthol green B biodegradation	[149]
Ag_2_S	NPs (53.4 nm for wild-type strain, 27.6 nm for*△mtrC-omcA* strain)	*S. oneidensis* MR-1	Extracellular	Catalytic reduction of methylviologen	[77]
	Monodispersed and homogeneous spherical NPs (9 ± 3.5 nm)	*S. oneidensis* MR-1	Extracellular	Non-inhibitory and non-cytotoxic effect on bacteria and eukaryotic cell lines	[150]
CuS	Homogenous NPs (∼5 nm), high hydrophility and stablity	*S. oneidensis* MR-1	Extracellular	Photothermal agent	[152]
	Hollow CuS nano/micro shell (diameter of 17.4 nm, length of 80.8 nm)	*S. oneidensis* MR-1	On cell surface	Cr^6+^ removal	[153]
ZnS	Spherical NPs (~ 5 nm)	*S. oneidensis* MR-1	Extracellular	Photodegradation of rhodamine B	[154]
Mn:ZnS	Nano quantum dots (5–10 nm)	*S. oneidensis* JG3631	Extracellular	–	[155]
CdS	NPs (15 nm)	*S. oneidensis* MR-1	Extracellular	Increased cytotoxic effect on brain cancer cell lines	[158]
	NPs (4.5–11.5 nm, average 7 nm)	*S. oneidensis* MR-1	On cell surface	Photoreductive degradation of trypan blue	[159]
	NPs	*G. sulfurreducens* PCA	On cell surface	Light-driven bio-decolorization of methyl orange	[30]
CdSe	Ultrafine NPs (3.3 ± 0.6 nm)	*S. oneidensis* MR-1	Inside cytoplasm	Yellow fluorescence	[160]
HgSe	Monodispersed NPs (4.3 ± 0.79 nm)	*S. putrefaciens* 200	On cell surface	–	[169]

### Extraction and purification of biogenic metal nanoparticles

The extraction, concentration and purification of the biologically produced MNPs directly determine their practical applications and commercial competitiveness. Centrifugation is undoubtedly one of the most effective strategies to separate and concentrate MNPs due to its simplicity, low cost and easy scalability. Apparently, the biosynthetic route based on bacterial EET ability provides a distinct advantage for centrifugal separation and purification because many metal ions could be reduced directly to from corresponding MNPs outside the cells by taking advantage of the electrons that are transported out of the cells. In this case, the generated MNPs could be collected directly by simple centrifugation process. Generally, a two-step centrifugation strategy with different centrifugal forces was adopted: the low one (~ 5000 g) for firstly removing bacterial cells and the high one (up to 100,000 g) for collecting MNPs subsequently [20, 21]. Besides, filter units were also applied to effectively concentrate MNPs from a large-volume reaction solution [161]. For extraction and purification of those MNPs either inside the cells or attached on cell surfaces, the release of them from bacterial cells through physical or biological disruption of cells (e.g. ultrasonic collapsing, autoclaving and lysozyme lysis) is unavoidable before centrifugal collection [90, 148]. Remarkably, given the inherent and interesting features of *Shewanella* and *Geobacter* species themselves, a lot of studies have attempted to directly use the MNPs-hybridized cells without additional extraction and purification of MNPs for both bioelectricity production and pollutant removal with satisfactory performance, which not only simplifies the production process but also effectively integrates the merits of inorganic nanoparticles and bacterial cells [24, 34, 69, 73, 146, 162, 163].

## Graphene oxide bioreduction

Graphene has been becoming a celebrity in material science since its revolutionary discovery by Novoselov and co-workers [170]. Graphene possesses ultrahigh specific surface area and many appealing mechanical, electrical, optical and thermal properties [171]. Chemical oxidation of graphite to graphene oxide (GO) followed by the reduction treatment is a commonly used method for producing graphene [172]. Properties of the reduced graphene oxide (RGO) are dependent to a large extent on reductants and reduction conditions. Using chemical reductants such as hydrazine is an efficient approach with respect to large-scale production at a low cost, but many of them are potentially toxic to living organisms. In addition to use green reducing agents such as ascorbic acid [173], microbial reduction is an alternative strategy.

Five strains of *Shewanella* species were investigated by Salas et al. on the biological reduction activities towards the production of GO under anaerobic conditions, where the reduction was evident at 24 h for all of the bacteria although in varying degrees [31]. Impressively, physical characteristics such as conductivity of bacterially reduced GO were comparable to the chemically reduced counterpart, and the EET pathway of *S. oneidensis* MR-1 was demonstrated to play a significant role in the GO reduction because of an apparently impaired reduction ability with those strains deficient in each of outer membrane *c*-Cyts, but there is a puzzling finding in their work that the *△cymA* mutant retained an almost unscathed ability in the GO reduction [31]. Soon after, Jiao et al. reported a quite contrary experimental phenomenon that the mutated *S. oneidensis* MR-1 lack of *cymA* gene was incapable of reducing GO [174]. Moreover, Yu and co-workers found that the kinetics of electron transfer from the purified protein OmcA to GO was obeyed the Michaelis–Menten equation, indicating that the electron transfer process was assigned to an enzyme catalytic characteristic [175]. Their further study on structure of OmcA/GO complex suggested that the formation of a hydrogen bond between the -NH_2_ group of amino acid residues and the -COOH/-OH group of GO could shorten the interfacial electron transfer distance so as to mitigate the energy barrier. This work gives substantial evidences at the molecular level about bacterial *c*-Cyts mediated GO reduction.

In addition, electron-shuttling compounds such as riboflavin and AQDS can increase the GO reduction rate by several times because of their high electron-carrying capacities [174, 176]. Notably, the microbial reduction of GO was also observed in spite of the presence of oxygen, which meant that the strict anaerobic environment is not essential although that oxygen molecules are more favorable electron acceptors than GO [177, 178]. The occurrence of GO reduction under aerobic conditions might be ascribed to either an oxygen-lacking microenvironment where the oxygen was non-accessible or/and exhausted [177], or the chemical reduction executed by some specific biomolecules [178].

Assembly of 2-D graphene sheets serving as building blocks into 3-D architectures is extremely attractive because of their unique structure features with broad application range [179]. During the microbial reduction process, biogenic RGO can self-aggregate into 3-D conductive hydrogel complex together with bacterial cells, and the produced complex is usually referred to as hybridized biofilms [32, 180, 181]. Compared to those chemically assembled 3-D graphene architectures, the biological counterparts provide an elastic platform to integrate conductive graphene with luxuriant biological functions of bacteria.

It is noteworthy that the 2-D sheet structure is a promising host for the deposition of diverse nanoparticles. On account of the confirmed ability of DMRB to reduce both metal ions and GO, green and facile one-pot synthesis of functional hybrid nanomaterials consisting of MNPs and RGO can come true under an ambient condition. Dong et al. developed an Au-NPs/RGO nanohybrid synthesized by using *S. oneidensis* MR-1 without the addition of any toxic agents, which showed comparable structural features and a better catalytic activity towards the reductive removal of nitroaromatics [182]. The similar approaches have also been adopted to biosynthesize Ag-NPs/RGO [164], Pd-NPs/RGO [183], Pd-Au/RGO[25] and Pd–Ag/rGO[168] nanocomposites.

All of above studies demonstrate a simple, eco-friendly and cost-effective methodology for the fabrication of graphene-based nanocomposites with particular functionalities and application potentials.

## Applications

### In-situ assembly of bioelectrodes

A bioelectrode wiring electroactive bacterial cells to conductive solid interface is the core footstone to develop diverse microbial electrochemical systems [184, 185]. Notably, how to strengthen the bio-abiotic interfacial electron transport has always been a critical challenge, because the low efficiency of the process severely impedes the practical applications of microbial electrochemical devices [13]. Assembly of such a bioelectrode enveloped with hybridized biofilms in which functional nanomaterials are biosynthesized in-situ by bacterial cells and thereby form a cooperative alliance together with the cells is a productive route to remarkably enhance interfacial electron transfer. Yong et al. reported a pioneering work on the self-assembly of GO and *S. oneidensis* MR-1 to form the 3-D electroactive RGO-hybridized biofilm where the GO nanosheets acted as fishing nets to catch bacteria cells and was reduced in turn by the captured cells [32]. Inspiringly, the developed electroactive biofilm delivered a 25-fold increase in the outward current (electron flux from bacterial cells to electrode) and 74-fold increase in the inward current (reversed electron flux) over that of the naturally occurring biofilm. The high incorporation of bacterial cells into the electrode and the enhanced direct EET pathway bridged by biogenic RGO were suggested to be responsible for the dramatical improvements. Afterwards the tactics of in-situ assembly of graphene/electroactive bacteria hybrid biofilms has been widely adopted to boost interfacial EET, leading to greatly improved performances of microbial electrochemical systems for bioelectricity production, carbon dioxide reduction and Cr^6+^ removal [186–188].

The in-situ incorporation of MNPs with fine conductivity and catalytic activity into electroactive biofilm is another emerging approach to aggrandize electrode performance, because the high-conductive nanoparticles attached on bacterial cells are expected to rescue the relatively poor conductivity of the cells. Inspired by this conception, Hou et al. constructed a 3-D conductive bio-network through in-situ synthesis of Pd-NPs by *G. sulfurreducens* PCA biofilm to improve the electron transfer, which achieved an over fivefold increase in both hydrogen evolution and the reductive degradation of nitro-, azo- and chloro-aromatics [189]. The incorporation of biogenic Au-NPs was also proved to greatly increase the conductivity of *G. sulfurreducens* PCA biofilm, leading to 40% increase in anodic current density [73]. To further break through the challenge of sluggish electron transfer between biosynthesized nanoparticles and an electrode, a ternary hybrid biofilm of Pd-NPs/cells/RGO was developed recently through the simultaneous reduction of Pd^2+^ and GO using *S. oneidensis* MR-1 [190]. Compared to the binary control without RGO, electrochemical conductivity of the prepared Pd-NPs/cells/RGO hybrid biofilm increased from almost zero to 196 μS cm^−1^ because of the well-developed 3-D electron transfer network with the implantation of RGO. As a consequence, the ternary biofilm showed outstanding electrocatalytic activity in terms of 36.7- and 17.2-fold increase in steady state current density towards hydrogen evolution and nitrobenzene reduction, respectively.

**Fig. 7:**
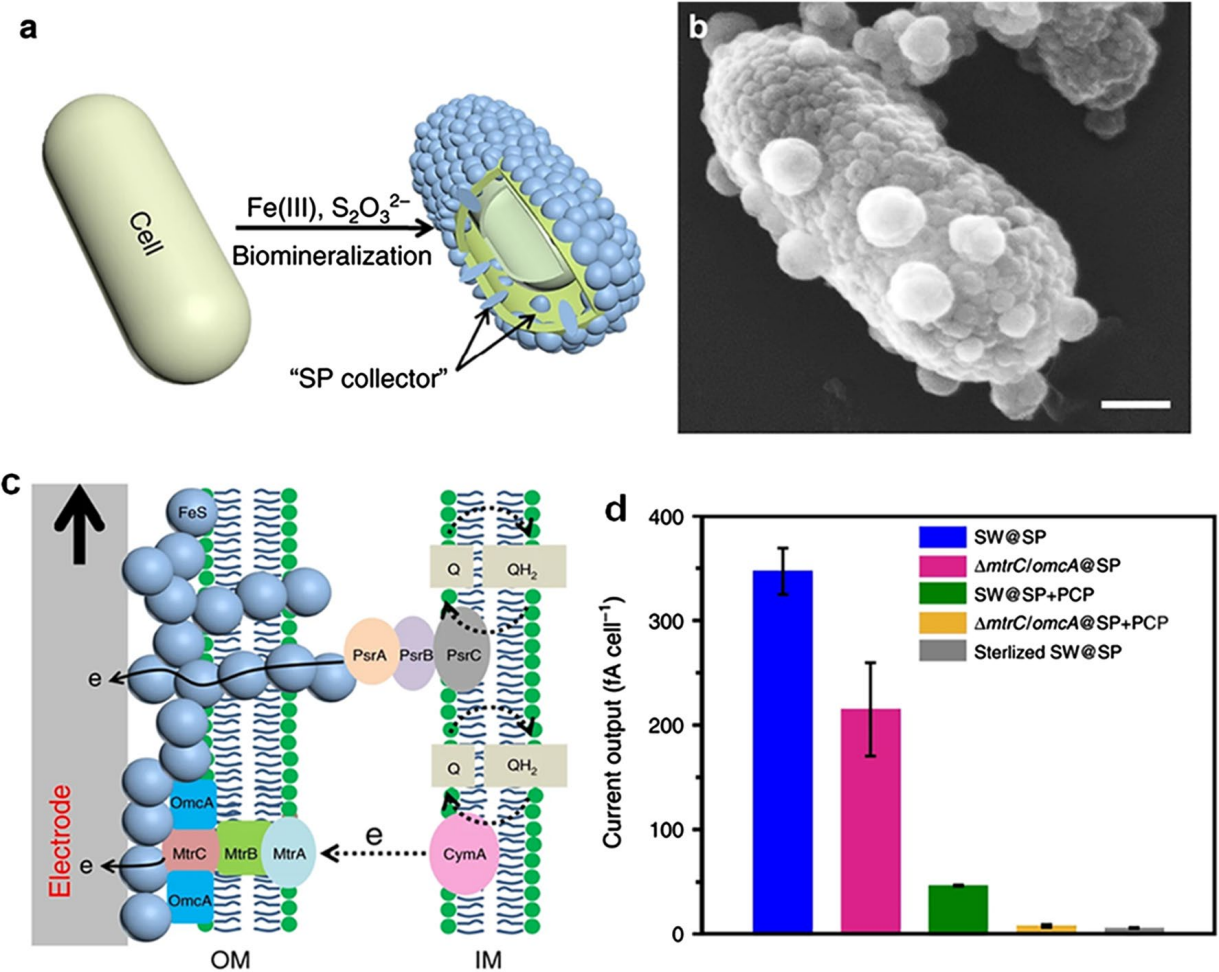
A single cell electron collector in-situ assembled with biogenic FeS-NPs for significantly increasing interfacial electron transfer efficiency at the single-cell level and electricity production. **a** Schematic illustration of the FeS-NPs-based collector assembly, **b** SEM image of *S. oneidensis* MR-1 cell coated with FeS-NPs, **c** proposed electron transfer pathway from the cell as the electron collector to an electrode, **d** current output of the cell under different conditions [34]

Except for noble metal nanoparticles, nanosized FeS biosynthesized during the *Shewanella* biofilm maturation was demonstrated to significantly enhance bioelectricity production as well [143, 144], where the biogenic FeS-NPs were regarded as extracellular electron conduits wiring the electron-producing cells to the solid electrode. Very recently, Yu and co-workers proposed a new concept of a single cell electron collector, which was in-situ built with an interconnected intact conductive layer on and cross an individual cell membrane [34]. The single cell electron collector assembled with biogenic FeS-NPs was proved to achieve a record-high interfacial electron transfer efficiency and electricity production (Fig. 7). The improvement could be attributed to the fact that the FeS-NPs wrapped around cell surface of *S. oneidensis* MR-1 wired the MtrC/OmcA-MtrB-MtrA transmembrane electron conduits to electrode, while others in the periplasm bridged the periplasm-terminated conduits such as polysulfide reductase PsrABC (a bio-complex responsible for FeS biosynthesis). This innovative work opens a new window for abiotic/biotic interface engineering to improve interfacial electron transfer efficiency from macro-population levels to single-cell levels.

### Nano-catalysts for energy conversion

Nanostructured metal materials especially noble metals are the most commonly used catalysts for various energy conversion reactions because of their incomparable reactivities [191]. Not surprisingly, biogenic MNPs synthesized by DMRB have also cut a figure in this area.

As one case, nano-sized Pd particles produced by *S. oneidensis* MR-1 cells have been exploited as effective oxygen reduction catalysts for fuel cells and metal-air batteries [166]. After simple KOH activation at 420 °C, *S. oneidensis* MR-1 cells were converted into highly porous heteroatom-doped carbon supporting uniform Pd-NPs, and consequently, the as-prepared hybrid nanomaterials showed 2.2-fold higher specific mass catalytic activity, better durability and methanol tolerance compared to the commercial Pt/C catalyst. Recently, Wang et al. synthesized a Pd/RGO hybrid catalyst through using *S. oneidensis* MR-1 as the biological reducing agent, which showed promising electrocatalytic activity towards oxygen reduction reaction in alkaline electrolyte [183]. Jiang and co-workers fabricated another efficient oxygen reduction catalyst of Mn_2_O_3_ micro-/nanocubes through calcination of biogenic MnCO_3_ precursors that were produced by *S. loihica* PV-4 in the presence of MnO_4_^−^ as the sole acceptor [192].

The bacteria-derived MNPs have also been tried as Li-ion storage materials. For instance, *S. oneidensis* MR-1 was reported to synthesize Te nanorods made up of helically-twisted atomic-wire bundle structure, which gave an unique Li-ion uptake characteristic [98]. The thermal carbonization of these biogenic Te nanorods together with bacterial cells provided a well-defined encapsulation of Te nanorods into carbon matrix, leading to an increased electrical conductivity and enhanced battery performance. More notably, these biogenic Te nanorods behaved the reversible Li-ion uptake without structural deterioration owing to their unusual anionic redox chemistry and structural flexibility. For another example, As_4_S_4_ clusters with unique molecular-cage-like structure produced by *Shewanella* sp. HN-41 were adopted as high-performance Li-ion active storage materials [141].

In general, the biogenic MNPs cannot be used directly as electrocatalysts because of the coexistence of poor-conductive cell substrates. Therefore, a high-temperature carbonization approach mentioned above are usually involved in the preparation process [98, 166]. As an alternative, a simple hydrothermal reaction has been also proposed to convert non-conducting cell biomass into heteroatom doped carbon matrix with porous structure and high conductivity. For instance, a 3D porous bio-PdAu/RGO catalyst was harvested through a facile hydrothermal treatment of bacteria/PdAu/GO hybrid biofilm, which exhibited a better electrocatalytic activity and durability towards oxidation of both ethanol (alkaline condition) and formate (acidic condition) compared to a commercial Pd/C [25].

Noteworthily, the bacterial biomass is a kind of versatile precursors with a distinguishing feature of in-situ heteroatom doping, which is expected to open up a horizon into elastically tailoring the characteristics of biogenic catalysts.

### Organic pollutant degradation

As showed in Table 1, numerous biogenic MNPs and their derivatives have been used widely as nanocatalysts for degradation of various organic pollutants, especially 4-chlorophenol and azo dyes. For instance, Au-NPs biosynthesized by *S. haliotis* were found to behaved size- and shape-dependent catalytic activity towards 4-nitrophenol reduction using sodium borohydride (NaBH_4_) as chemical reductants, of which the spherical and small particles gained the highest activity with a rate constant of 0.665 min^−1^ [69]. Another, the bio-Pd/Pt alloy nanoparticles produced by *S. oneidensis* MR-1 also showed high activity to 4-nitrophenol reduction, while there was a gradual downturn of catalytic activity [26].

Controlling the particle size distribution and preventing the unfavorable aggregation of nanoparticles in the process are of importance for their activities and durabilities in practical applications. Carbon nanomaterials having high surface area, conductivity and stability are very promising to address this concern. Various biogenic MNPs including Ag-NPs [193], Cu-NPs[105] and Pd/Ag alloys[168] have been integrated into either carbon nanotubes or graphene as efficient catalysts for organic contaminant removal. Additionally, the conversion of bacterial cell biomass to porous carbon matrix through KOH activation at a high temperature was also developed to improve the catalytic activity of biogenic Pd-NPs [22]. The as-activated catalysts delivered a high-performance catalytic reduction of 4-nitrophenol to 4-aminophenol with an outstanding apparent kinetic constant of 5.0 × 10^–3^ s^−1^, comparable to the commercial Pd/C (5.0 wt%). Biogenic FeS-NPs synthesized by *Shewanella* spp. have been reported to dramatically accelerate dechlorination of both carbon tereachloride and trichloroethylene by a factor of over five compared to the abiotic FeS [147].

**Fig. 8:**
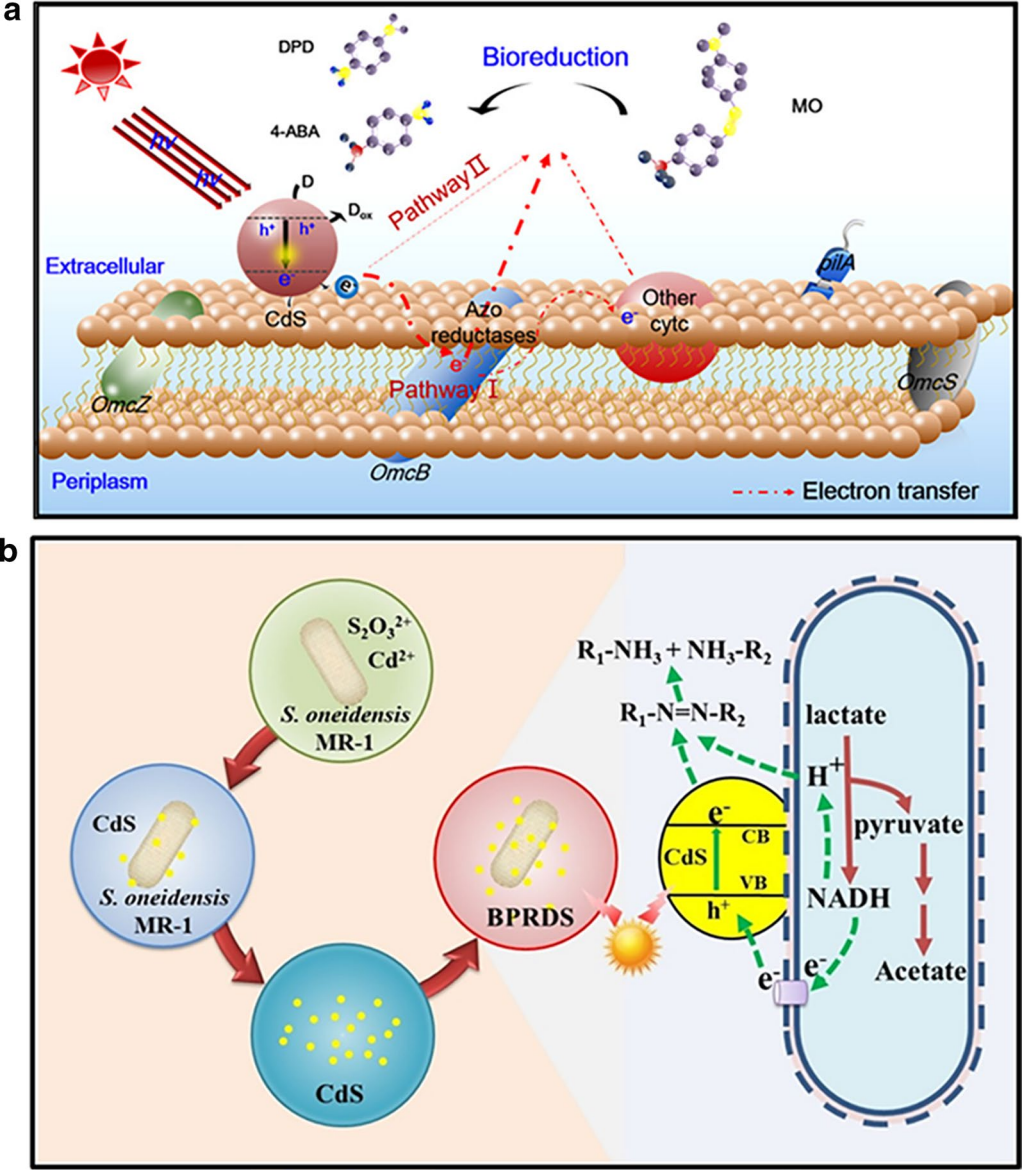
Bio-photo-catalytic systems produced through in-situ assembling biogenic CdS quantum dots into cells. **a** CdS-hybridized *G. sulfurreducens* PCA for bio-photo-catalytic degradation of methyl orange (MO).[30] **b** CdS-hybridized S*. oneidensis* MR-1 for bio-photo-catalytic degradation of trypan blue [159]

The application of biogenic MNP-based catalysts has indeed made great progress in assisting the chemical degradation of organic pollutants, while such methodology usually needs to use toxic chemicals as reductants. Both photo-catalytic and bio-catalytic degradation without the use of harmful reagents are much more favorable. Impressively, the in-situ formation of biogenic CdS quantum dots on the cell surface of *G. sulfurreducens* PCA and S*. oneidensis* MR-1 provided an opportunity to develop an attractive hybrid pattern that integrated biological degradation with ligh-excited photoelectrons (Fig. 8) [30, 159]. Such a bio-photo-catalytic system opened a new high-powered strategy for pollutant degradation through interactions among light energy, electrochemical reactions and microorganisms. Besides, biosynthesized Pd-NPs and RGO were incorporated into *S. oneidensis* MR-1 biofilm for significantly improving bioelectrochemical removal of nitrobenzene, because these biogenic nanomaterials constructed a fast electron transfer network in electrode biofilms [190]. Observably, the development of biohybrid systems together with biogenic nanomaterials holds great competitiveness in pollutant degradation.

### Heavy metal removal and recycling

Heavy metal pollution poses serious threat to both human health and environmental safety. In contrast to organic pollutants that can be effectively degraded, heavy metals can only be treated by adsorption separation or/and (bio-)chemical transformation that produce insoluble precipitates with less toxicity. Not only can microorganisms serve as bio-adsorbents for heavy metals, but also drive their bio-transformation for detoxication [157]. It is well known that DMRB can efficiently reduce various metal ions with relatively high redox potentials under anaerobic conditions, and consequently transform toxic heavy metal ions into functional nanomaterials.

As an important complement to microbial recycling, the application of biogenic nanomaterials especially MNPs for the detoxification of heavy metal pollution is also illuminated recently. Hexavalent chromium (Cr^6+^), one of the most prevalent heavy metal contaminants, is approximately 100 folds more toxic and 1000 folds more mutagenic than its reduced form of Cr^3+^ with much less solubility and mobility in aqueous environment [194], and thus the reductive transformation of Cr^6+^ to Cr^3+^ is a generally approving way to remediate Cr^6+^ pollution. Biogenic Pd-NPS produced by both *Shewanella*[163] and *Geobacter* species[162] exhibited high removal capacities for Cr^6+^. The nanostructured FeS is another attractive candidate for Cr^6+^ reduction since both Fe^2+^ and S^2−^ are excellent reductants. The feasible removal of aqueous Cr^6+^ pollution by biogenic FeS-NPs was reported by Yu et al. and the removal efficiency was dependent on the particle size [148]. Taking into consideration of their inherent abilities to biological reduce metal ions, the DMRB cells hybridized with biogenic MNP are very expected to remove toxic metal pollution due to the potential merit of bio-abiotic interactions [162].

Nanoscale magnetite is also an attractive material for removing toxic heavy metals due to its especial magnetic recoverability [131]. Impressively, the biogenic magnetite nanoparticles achieved a higher Cr^6+^ removal efficiency (almost 100%) than the chemically synthesized counterpart (82%), because the negative-charged organic coating could effectively enhance electrostatic adsorption of positive-charged chromium ions for the former [195].

### Antimicrobial activity and therapy

Ag-NPs are considered promising for combating bacteria due to their low cytotoxicity and remarkable antimicrobial activity against both Gram-positive and Gram-negative bacteria [196]. Much effort has been devoted to chemically synthesize shape-controllable Ag-NPs for pharmaceutical applications. Nevertheless, biogenic Ag-NPs synthesized by *S. oneidensis* MR-1 were found to present stronger toxicity for all targeted bacterial strains including *E. coli*, *S. oneidensis* and *B. subtilis* compared to chemically synthesized ones such as colloidal-Ag and oleate capped Ag-NPs [21]. The nanoparticle surface coatings appear to play a critical role in the antimicrobial activity of biogenic Ag-NPs, while further investigations are needed to explore underlying mechanism. The antibacterial activity of Ag-NPs seemed size-dependent, and increased with a decrease in the particle diameter [77]. A biogenic Ag/RGO hybrid, the *Shewanella*-synthesizing Ag-NPs with a diameter < 10 nm growing on RGO without aggregation, showed an excellent sterilization capacity up to 99.9% against *E. coli* when used at a dosage of 2 mg/L for 15 min, and the rapid release of Ag^+^ to enhance the bacteria-bactericide interaction and the strong convolution of bacterial cells by RGO were considered to make contributions to the high antibacterial activity [164]. Biogenic Cu-NPs[104] and Te-NPs[96] also showed antibacterial activity against several pathogenic bacteria.

Biogenic MNPs are also attracting widespread interest in their applications in other biomedical fields. Nanostructured CuS, one kind of cheap semiconductors with strong and stable absorption in near infrared region, is a promosing candidate for photothermal therapy. Zhou et al. fabricated homogenous CuS-NPs with small size (~ 5 nm) by *S. oneidensis* MR-1 and evaluated their performance in photothermal therapy for the first time [152]. The biogenic CuS-NPs showed a high photothermal conversion efficiency of 27.2% due to their strong absorption of the infrared irradiation, indicating their potentials as a photothermal therapy agent. As another example, CdS-NPs produced by *S. oneidensis* MR-1 with ionic liquid as soft template exhibited excellent cytotoxicity against brain cancer cell lines using rat glioma cell lines [158].

## Conclusions and perspectives

The potential use of nanosized materials in various areas triggers the increasing need to produce them in stable and tailorable formulations with environmental-friendly processes. There is therefore ongoing research for implementing biotechnological and green synthesis methodologies. Microorganisms, as powerful biological nanofactories, have proven themselves capable of rapidly synthesizing various nano-scale materials, especially MNPs.

*Shewanella* and *Geobacter* species with specific EET pathways are competitive over other microorganisms in controllable synthesis of MNPs with well-defined sizes and structures, since they can directly synthesize MNPs through extracellular reduction without a need for transporting metal ions into the cells. Furthermore, the extracellular dissimilatory reduction abilities of *Shewanella* and *Geobacter* species allow them to produce biological RGO and hybrid materials together with various MNPs. Indeed, both direct EET via membrane-bound *c*-Cyts (Mtr proteins for *Shewanella* and Omc proteins for *Geobacter*) and indirect EET mediated by self-secreted electron shuttles like flavins have been evidenced to transport intracellular electrons (reducing equivalents) across the cell membrane barriers for the reduction of metal ions ot/and GO outside the cells, thus resulting in the formation of inoganic nanomaterials. Apart from the well-controlled nanostructures and physicochemical properties, the bacterial EET-driven synthesis route has also been illustrated substantially to make nanoproducts more biocompatible than the chemically produced counterparts in general, because such biological methodology adpotes biological constituents (e.g. proteins and bio-active small molecules) instead of chemcial reagents (e.g. reducing, capping and stabilizing agents) that are usually required for the chemical synthesis. The biogenic nanomaterials with non- or low-cytotoxicity are of great promise in biomedical application. It is predictable that the biosynthestic route is competitive economically in view of the low-cost and renewbale bacterial cells acting as nanofabricating biofactories and the fast biosynthesis rate (the time required for biosynthesizing MNPs could be as low as several minutes). What's more, the biological synthesis gives a promise to self-assemble inorgnic/biotic hybrid systems through in-situ formation of inorgnic nanomaterials interfacing with bactterial cells, which not only provides a new platform to study the biotic/abiotic interfacial interaction but also broadens the application range from classical areas (e.g. antibacterial and inorganic catalysis) to some emerging interdisciplinary disciplines such as bioelectrocatalysis and biophotocatalysis.

Although achieving great advances in nanobiosynthesis using *Shewanella* and *Geobacter* species as cell factories, there is still much to do to accurately tune sizes, nanostructures and properties of the biogenic nanoproducts for designated applications. In-depth elucidation and control of the EET route is beyond all doubt the key to achieve this goal. Over the past decade, studies on mechanisms underlying the bacterial EET of *Shewanella* and *Geobacter* species have made substantial progress using solid electrodes electron acceptors, but an understanding regarding how the EET pathways take part in the formation of inorganic nanoparticles is still in its infancy. Clear bioformation on the EET pathways is the foundation of controlling the sizes, shapes, locations and dispersities of nanoproducts, and advanced omics technologies such as differential proteomics combined with genomics will be helpful in identifying the key proteins or/and electron transport routes involved in the nanoparticle biosynthesis for the design and construction of new biological entities under the guidance of synthetic biology strategy to produce custom-tailored nanomaterials. Noteworthily, the artificial construction of biological/inorganic hybrids that tactfully combine functional inorganic nanomaterials with bacterial vitalities are gaining great popularity.

Nanobiotechnology is highly interdisciplinary, which requires collaboration between different sciences including biology, nanoscience, materials science, chemistry, etc. We envision that with continuous progresses in the biosynthesis mechanisms and biotic/abiotic techniques, the versatile EET feature of microorganisms will unfold greater success in the biosynthesis of inorganic nanomaterials and their applications. The feasibility for the biological production of MNPs on industrial scales is also a crucial point to be considered, in particular their extracellular production, which is of great significance for product recovery. So far, only a few studies have reported the scale-up production of biogenic MNPs. As a result, how to increase the productivity of extracellular MNPs needs more attention in the future.

## Data Availability

Without restrictions.
